# Author Correction: MerMAIDs: a family of metagenomically discovered marine anion-conducting and intensely desensitizing channelrhodopsins

**DOI:** 10.1038/s41467-024-48358-2

**Published:** 2024-05-10

**Authors:** Johannes Oppermann, Paul Fischer, Arita Silapetere, Bernhard Liepe, Silvia Rodriguez-Rozada, José Flores-Uribe, Enrico Schiewer, Anke Keidel, Johannes Vierock, Joel Kaufmann, Matthias Broser, Meike Luck, Franz Bartl, Peter Hildebrandt, J. Simon Wiegert, Oded Béjà, Peter Hegemann, Jonas Wietek

**Affiliations:** 1 Institute for Biology, Experimental Biophysics, Humboldt-Universität zu Berlin, Invalidenstraße 42, 10115 Berlin, Germany; 2 Research Group Synaptic Wiring and Information Processing, Center for Molecular Neurobiology Hamburg, Falkenried 94, 20251 Hamburg, Germany; 3 Technion—Israel Institute of Technology, 32000 Haifa, Israel; 4 Institute for Chemistry, Physical Chemistry/Biophysical Chemistry, Technische Universität Berlin, Straße des 17. Juni 135, 10623 Berlin, Germany; 5 Institute for Biology, Biophysical Chemistry, Humboldt-Universität zu Berlin, Invalidenstraße 42, 10115 Berlin, Germany; 6 Present Address: Department of Plant Microbe Interactions, Max Planck Institute for Plant Breeding Research, Cologne, 50829 Germany; 7 Present Address: Department of Neurobiology, Weizmann Institute of Science, 7610001 Rehovot, Israel

Correction to: *Nature Communications* 10.1038/s41467-019-11322-6, published online 25 July 2019

Since the publication of this work, Enrico Schiewer has changed their name from Enrico Peter. This has now been amended in the HTML and PDF versions of the article.

