# Supplementary material for: MerMAIDs: a family of metagenomically discovered marine anion-conducting and intensely desensitizing channelrhodopsins
Source: Nat Commun. 2019 Jul 25;10:3315. doi: 10.1038/s41467-019-11322-6 (PMC6658528; doi:10.1038/s41467-019-11322-6)
Supplement: Supplementary file 2 — Reporting Summary [file 41467_2019_11322_MOESM2_ESM.pdf]

nature research

Corresponding author(s): Peter Hegemann, Jonas Wietek

Last updated by author(s): 2019-19-06

Reporting Summary

Nature Research wishes to improve the reproducibility of the work that we publish. This form provides structure for consistency and transparency in reporting. For further information on Nature Research policies, see Authors & Referees and the Editorial Policy Checklist.

Statistics

For all statistical analyses, confirm that the following items are present in the figure legend, table legend, main text, or Methods section.

n/a	Confirmed	
☐	☒	The exact sample size (n) for each experimental group/condition, given as a discrete number and unit of measurement
☐	☒	A statement on whether measurements were taken from distinct samples or whether the same sample was measured repeatedly
☐	☒	The statistical test(s) used AND whether they are one- or two-sided<br>*Only common tests should be described solely by name; describe more complex techniques in the Methods section.*
☒	☐	A description of all covariates tested
☒	☐	A description of any assumptions or corrections, such as tests of normality and adjustment for multiple comparisons
☐	☒	A full description of the statistical parameters including central tendency (e.g. means) or other basic estimates (e.g. regression coefficient) AND variation (e.g. standard deviation) or associated estimates of uncertainty (e.g. confidence intervals)
☐	☒	For null hypothesis testing, the test statistic (e.g. F, t, r) with confidence intervals, effect sizes, degrees of freedom and P value noted<br>*Give P values as exact values whenever suitable.*
☒	☐	For Bayesian analysis, information on the choice of priors and Markov chain Monte Carlo settings
☒	☐	For hierarchical and complex designs, identification of the appropriate level for tests and full reporting of outcomes
☒	☐	Estimates of effect sizes (e.g. Cohen's d, Pearson's r), indicating how they were calculated

Our web collection on statistics for biologists contains articles on many of the points above.

Software and code

Policy information about availability of computer code

Data collection	Experimental data was collected using software follows: Electrophysiological data from HEK cells (Clampex 10.4, Molecular Devices, Sunnyvale, CA), stationary UV/vis spectral data (UVProbe v2.34, Shimadzu, Kyōto, Japan and Varian UV v3.0, Varian, Mulgrave, Australia), single-turnover transient absorption spectral data (custom software written in visual C++), Raman spectral data (LabSpec Spectroscopy Suite software, Horiba, Villeneuve, France), FTIR data (OPUS 7.5 software, Bruker Optics, Karlsruhe), UV/vis data obtained from FTIR samples (custom written in C#), electrophysiological data recorded from neurons (SutterPatch 2.0, Sutter Instrument, Novato, CA) and imaging data (ScanImage 2017b, Vidrio Technologies, Ashburn, VA).
Data analysis	Experimental data was analyzed with following software: Electrophysiological data from HEK cells (Clampfit 10.4, Molecular Devices), stationary UV/vis spectral data (Origin 2017, OriginLab, Northampton, MA), single-turnover transient absorption spectral data (MATLAB R2016b, The MathWorks, Natick, MA and Glotaran 1.5.1, Snellenburg et al. 2012), Raman spectral data (LabSpec Spectroscopy Suite software, Horiba and custom written spectra processing package), FTIR data (OPUS 7.5 software, Bruker Optics and custom code implemented in Octave 4.2.), UV/vis data obtained from FTIR samples (custom code in Octave 4.2. and MATLAB R2016b), electrophysiological data recorded from neurons (Igor Pro 8.0, wavemetrics, Lake Oswego, OR) and imaging data (ScanImage 2017b, Vidrio Technologies and Fiji, Schindelin et al. 2012).

For manuscripts utilizing custom algorithms or software that are central to the research but not yet described in published literature, software must be made available to editors/reviewers. We strongly encourage code deposition in a community repository (e.g. GitHub). See the Nature Research guidelines for submitting code & software for further information.

Data

Policy information about availability of data

All manuscripts must include a data availability statement. This statement should provide the following information, where applicable:

- Accession codes, unique identifiers, or web links for publicly available datasets
- A list of figures that have associated raw data
- A description of any restrictions on data availability

The source data underlying Figs 1d-g, 2b-d,e-g,i,j, 4e-i and Supplementary Figs 3b,e, 4a-l, 5d,h are provided as a Source Data file . Code and data of metagenomic analysis can be obtained from https://github.com/BejaLab/MerMAIDs. Plasmids for mammalian codon-optimized expression of MerMAID1-7 are available at addgene (addgene ID 126513-19, respectively). Plasmids for AAV production of MerMAID1,6 are available at addgene IDs 126520, 126521, respectively. GenBank information of MerMAID1-7 can be obtained under accession codes MK914541-7, respectively. Further data, material or code is available from the corresponding authors upon reasonable request.

Field-specific reporting

Please select the one below that is the best fit for your research. If you are not sure, read the appropriate sections before making your selection.

☒ Life sciences ☐ Behavioural & social sciences ☐ Ecological, evolutionary & environmental sciences

For a reference copy of the document with all sections, see nature.com/documents/nr-reporting-summary-flat.pdf

Life sciences study design

All studies must disclose on these points even when the disclosure is negative.

Sample size	No statistical tests were used to predetermine sample size. Sample sizes were similar to those commonly used in this research field.
Data exclusions	Electrophysiological data from HEK cell recordings with access resistance >10 MOhm and membrane resistance <1 GOhm were excluded from analysis.
Replication	Electrophysiological recordings from HEK cells were repeated (multiple cells from multiple transfection batches) and always refer to biological replicates.
Randomization	Randomization was performed in case of buffer exchange experiments and automated analysis was used whenever possible.
Blinding	Blinding was not performed to ensure correct assignment of the data to the measured constructs and/or experimental conditions.

Reporting for specific materials, systems and methods

We require information from authors about some types of materials, experimental systems and methods used in many studies. Here, indicate whether each material, system or method listed is relevant to your study. If you are not sure if a list item applies to your research, read the appropriate section before selecting a response.

Materials & experimental systems

n/a	Involved in the study	
☒	☐	Antibodies
☐	☒	Eukaryotic cell lines
☒	☐	Palaeontology
☐	☒	Animals and other organisms
☒	☐	Human research participants
☒	☐	Clinical data

Methods

n/a	Involved in the study	
☒	☐	ChIP-seq
☒	☐	Flow cytometry
☒	☐	MRI-based neuroimaging

Eukaryotic cell lines

Policy information about cell lines

Cell line source(s)	HEK-293 cells (ECACC 85120602, Sigma-Aldrich, Munich, Germany)
Authentication	Cell line was authenticated by vendor.
Mycoplasma contamination	tested by vendors and routinely tested by DAPI stain.

Commonly misidentified lines (See ICLAC register)	n/a

Animals and other organisms

Policy information about studies involving animals; ARRIVE guidelines recommended for reporting animal research

Laboratory animals	Female Wistar (Hsd:WI) rats (Envigo) were used for slice cultures.
Wild animals	The study did not involve wild animals.
Field-collected samples	The study did not involve samples collected from the field.
Ethics oversight	Animal procedures were in accordance with the guidelines of local authorities and Directive 2010/63/EU.

Note that full information on the approval of the study protocol must also be provided in the manuscript.
