# Supplementary material for: MerMAIDs: a family of metagenomically discovered marine anion-conducting and intensely desensitizing channelrhodopsins
Source: Nat Commun. 2019 Jul 25;10:3315. doi: 10.1038/s41467-019-11322-6 (PMC6658528; doi:10.1038/s41467-019-11322-6)
Supplement: Supplementary file 4 — Description of Additional Supplementary Files [file 41467_2019_11322_MOESM4_ESM.docx]

**Title:** Supplementary Data 1
**Description:** Overview of ChRs used to generate the phylogenetic tree in Fig. 1a and the sequence alignment in Supplementary Fig. 2. For previously described ChRs, AA IDs, GenBank IDs, and PMIDs are listed. For MerMAIDs, the SAMEA (SAM, BioSample accession; E, EBI; A, Assay Sample) reads are listed. The first seven digits are the European Nucelotide Archive (ENA) EMBL sample ID. The second seven digits indicate the contig number of the sample. In addition, MerMAID GenBank IDs are listed.
